# Fair funding for mental health research

**DOI:** 10.1177/00048674231177226

**Published:** 2023-05-26

**Authors:** Alison R Yung, Marko Milicevic, Michael Berk

**Affiliations:** 1 Institute for Mental and Physical Health and Clinical Translation (IMPACT), Deakin University, Geelong, VIC, Australia; 2 Orygen and Centre for Youth Mental Health, The University of Melbourne, Parkville, VIC, Australia; 3 School of Health Sciences, The University of Manchester, Manchester, UK

Mental health and substance use disorders account for 12.7% of total burden of disease and 23.7% of the chronic non-fatal burden of disease in Australia, second only to musculoskeletal disorders (24.3%) (Australian Institute of Health and Welfare, 2021). Despite this high burden, funding for mental health research in Australia is low, at less than half of what would be expected relative to the disability and mortality of mental ill health (see Table 1).

**Table 1.** Government funding for mental health research 2016–2021.

		2016	2017	2018	2019	2020	2021	Total
NHMRC	Number of grants	1056	1103	1045	857	741	799	5601
	Number of MH grants	97	106	94	65	81	62	505
	Total funding	$828,821,760	$877,678,691	$783,269,192	$923,247,229	$762,465,859	$988,806,640.49	$5,164,289,372
	Total MH funding	$74,484,664	$85,594,843	$75,616,750	$71,484,142	$87,151,120	$86,765,685	$481,097,204
	% of funding to MH	8.99%	9.75%	9.65%	7.74%	11.43%	8.77%	9.32%
MRFF	Number of grants	–	79	126	208	203	46	662
	Number of MH grants	–	8	14	23	15	9	69
	Total funding	–	$91,697,620	$265,109,326	$738,821,290	$666,971,203	$93,443,125	$1,856,042,564
	Total MH funding	–	$3,096,864	$39,342,069	$43,498,435	$41,125,987	$18,709,582	$145,772,937
	% of funding to MH	–	3.38%	14.84%	5.89%	6.17%	20.02%	7.85%
Combined	Number of grants	1056	1182	1171	1065	944	845	6263
	Number of MH grants	97	114	108	88	96	71	574
	Total funding	$828,821,760	$969,376,311	$1,048,378,518	$1,662,068,519	$1,429,437,062	$1,082,249,765.49	$7,020,331,936
	Total MH funding	$74,484,664	$88,691,707	$114,958,819	$114,982,577	$128,277,107	$105,475,267	$626,870,141
	% of funding to MH	8.99%	9.15%	10.97%	6.92%	8.97%	9.75%	8.93%

NHMRC: National Health and Medical Research Council; MRFF: Medical Research Future Fund; MH: mental health.

These data were assembled using search terms mental health, mental illness, depressi*, anxi*, psychosis, substance use, alcohol use, smoking, adhd, stimulants, cocaine, heroin, amphetamine, sleep, obsessive, panic, ptsd, suicide, bipolar, schizo*, illicit drug, autis*, psychiat* and trauma, as searched in publicly available funding results from the NHMRC and MRFF. These figures differ from official data, as funding bodies categorise mental health research as any research with a component that is relevant to mental health, and includes much fundamental neuroscience (Batterham et al.,2016), which was excluded from this search.

## Causes of under-investment

This underfunding is not due to poor quality research. Australian mental health researchers publish more than would be expected given funding and population size, and Australian mental health research performs better than research in oncology, endocrinology, cardiovascular disease and immunology in terms of rankings in international publications and citations (National Mental Health Commission, 2022). One explanation for the under-investment is that it is difficult, if not impossible, for mental health researchers to score highly on the National Health and Medical Research Council (NHMRC) rubric. For example, Table 2 shows the number of mental health projects that were considered to be in the top 10 ‘best’ projects for each year for 2013–2018. Only 4 out of 60 projects (6.67%) were mental health projects.

**Table 2.** NHMRC mental health projects listed in the 10 ‘best’ projects per year.

2013	0
2014	0
2015	0
2016	1
2017 (12th edition)	2
2018 (13th edition)^a^	1

NHMRC: National Health and Medical Research Council.

a Last available edition.

One criterion for scoring highly is ‘*has a scientific framework, design, methods and analyses that are flawless*’. Another is ‘*has or has access to exceptional technical resources, infrastructure, equipment and facilities*’. These are particularly hard to achieve for mental health research and can be considered from a PICOT (participants, intervention, control group, outcomes, timeline) framework.

### Participants

There is no clear pathology of any major mental health syndrome and there is a high degree of variation between participants with the same diagnosis. Such heterogeneity among participants may be seen as a weakness in study design by reviewers. Furthermore, for some psychiatric disorders the distinction between ‘normal’ emotions and illness is not clear. For example, in a study of depression, researchers may define a study group by using a cut-off on a scale. Reviewers may correctly note that someone just above the cut-off differs little from someone just below it.

Comorbidity is also an issue. Many individuals with one mental disorder also suffer from at least one other. Such comorbidity often creates problems in designing studies. Researchers could exclude individuals who have more than just the target diagnosis, but this creates an artificially small population from which to recruit, thus compromising feasibility, and results in a study group that is not representative of the overarching disorder. Alternatively, they need to adjust for comorbidity in the design or analyses. Such issues make a ‘flawless’ methodology unfeasible.

### Interventions

Instead of clear models of causality, mental health disorders result from multiple risk factors, which can vary greatly between individuals, and are often difficult to measure. Some may be unknown. It is thus not possible to write applications that have a neat link from the mechanism of an intervention to the targeted pathology and the resultant symptoms. Furthermore, mental health interventions are often complex. A psychological intervention could work via targeting behaviours, cognitions or by development of a therapeutic alliance. A double-blind design may be impossible. This means that compared to trials in general medicine, mental health studies cannot be ‘flawless’.

### Controls

When defining the control group, mental health researchers have a choice between including people with some symptoms that are below the threshold for disorder or excluding everyone with any psychiatric symptoms. In the former approach, controls may overlap with participants. They may have personality disorders and other symptoms that may impact the effectiveness of treatments and need to be considered in analyses. In the latter approach, controls may be ‘super-healthy’ with many protective factors and will not be representative of the general population. A further issue is the difficulty of recruiting controls that are like patient participants in socio-economic background and education, as mental health disorders are often more common in socially disadvantaged areas.

### Outcomes

Defining outcomes in mental health research is also more complex than in physical health research. For example, outcomes could be improvement of symptoms or functioning or adherence to medication. Ascertaining improvement in level of depression or quality of life (as examples) requires either researcher or participant judgement on a symptom scale. Compare this to a clearly defined 5-year survival metric that is a common outcome measure in cancer research.

### Timeline

Recruitment of participants for mental health studies is difficult, particularly in research focusing on severe and complex disorders. Ethics and consent issues may mean that the most unwell individuals are excluded from the study, thereby compromising generalisability and potential for translation. Long timelines are needed to ensure adequate recruitment, and thus studies may be expensive, and judged as ‘not good value for money’ by reviewers.

## Possible solutions

### Targeted funding

Chronic under-investment in mental health research has meant that there few clinical academics, limiting the capacity for growth of the sector. Services with a culture of research, in which clinicians and people with mental disorders are engaged in, and advocate for, studies and resources, are rare. There are few well-funded mental health research institutes, making ‘*access to exceptional technical resources, infrastructure, equipment and facilities*’ difficult. Specific funding to build capacity in mental health research would be one solution.

The NHMRC has a system for doing this. The Targeted Calls for Research (TCRs) are schemes that request grant applications to address a specific health issue and are ‘*designed to stimulate research or build research capacity in a particular area*’. They are an ideal mechanism for supporting and expanding mental health research capacity. Table 3 shows the number and proportion of funding awarded to mental health research from TCRs over the last 5 years. Again this number is not commensurate with the high burden of disease and prevalence of mental health disorders. We recommend more TCRs focused on mental health.

**Table 3.** Targeted Calls for Research awarded last 5 years (2017–2022).

	Number	%	Funding ($)	% of funding
Total	16	100	91,415,656	100
Mental health	2	12.5	10,557,597	11.6
Non-mental health	14	87.5	80,858,059	88.4

### Reviewers take burden of disease into consideration

Currently, the NHMRC instructs reviewers not to consider the prevalence or magnitude of the issue when reviewing applications. One change that would grow mental health research capacity would be to remove this instruction.

### Greater alignment of reviewer expertise to applications

This would ensure that reviewers were aware of the complexity of mental health research.

### ‘Relative to field of research’

When evaluating submissions for funding, the NHMRC has a ‘Relative to Opportunity’ policy (National Health and Medical Research Council, 2021) that aims to account for applicants’ career and available resources. We recommend a similar strategy in which assessment of applications would be indexed relative to what is feasible and that takes the technical challenges in the field into consideration. The NHMRC could also consider similar measures to the steps taken to improve gender equity (National Health and Medical Research Council, 2022) to promote equity between physical and mental health grants.

## Conclusion

Mental disorders are common and highly disabling. This needs to be reflected in a level of funding that reflects their substantial individual and societal impact. There are several practical avenues to achieve this goal.
